# Dual-Scale Porosity Alumina Structures Using Ceramic/Camphene Suspensions Containing Polymer Microspheres

**DOI:** 10.3390/ma15113875

**Published:** 2022-05-29

**Authors:** Hyun Lee, Jong-Won Jeon, Young-Hag Koh, Hyoun-Ee Kim

**Affiliations:** 1 Institute of Global Health Technology Research, Korea University, Seoul 02841, Korea; leeh0520@korea.ac.kr; 2 School of Biomedical Engineering, Korea University, Seoul 02841, Korea; rnxh125@naver.com; 3 Interdisciplinary Program in Precision Public Health, Korea University, Seoul 02841, Korea; 4 Department of Materials Science and Engineering, Seoul National University, Seoul 08826, Korea; kimhe@snu.ac.kr

**Keywords:** freeze casting, porogen, sacrificial templates, multi-scale porous ceramic

## Abstract

This study demonstrates the utility of thermo-regulated phase separable alumina/camphene suspensions containing poly(methyl methacrylate) (PMMA) microspheres as porogens for the production of multi-scale porosity structures. The homogeneous suspension prepared at 60 °C could undergo phase separation during freezing at room temperature. This process resulted in the 3D networks of camphene crystals and alumina walls containing PMMA microspheres. As a consequence, relatively large dendritic pores with several tens of microns size could be created as the replica of frozen camphene crystals. In addition, after the removal of PMMA microspheres via heat-treatment, micron-sized small spherical pores could be generated in alumina walls. As the PMMA content with respect to the alumina content increased from 0 vol% to 40 vol%, while the camphene content in the suspensions was kept constant (70 vol%), the overall porosity increased from 45.7 ± 0.5 vol% to 71.4 ± 0.5 vol%. This increase in porosity is attributed to an increase in the fraction of spherical pores in the alumina walls. Thus, compressive strength decreased from 153 ± 18.3 MPa to 33 ± 7.2 MPa. In addition, multi-scale porosity alumina objects with a honeycomb structure comprising periodic hexagonal macrochannels surrounded by dual-scale porosity walls were constructed using a 3D plotting technique.

## 1. Introduction

Porous ceramics can find very useful applications in diverse fields. For example, they can be used as lightweight structural components with high specific strengths and stiffness, scaffolds for bone regeneration [1,2,3,4], filters [5,6,7], thermal insulators [8,9], and electrical components [10,11,12]. Fundamentally, the characteristics of porous structures (i.e., porosity, pore size, pore geometry, pore interconnectivity, and pore size distribution) play key roles in the functions of porous ceramics [13]. In this regard, special attention has been paid to the creation of multiple pores at different length scales, in order to offer significantly enhanced mechanical functions at given porosities [14,15,16,17]. In particular, when used as bone scaffolds, macropores can provide favorable spaces for bone ingrowth and micropores in ceramic walls can stimulate bone regeneration [18,19,20].

In the manufacture of porous ceramics, the freeze casting of ceramic suspensions has demonstrated great advances, owing to its simplicity in processing and great ability to tailor the porosity and pore size [21,22,23,24,25,26]. More specifically, ceramic suspensions, comprising fine ceramic particles, freezing vehicle (e.g., water and camphene), and dispersant, are cast into molds below the melting point of the freezing vehicle. After this, the frozen objects are freeze-dried to remove frozen crystals. The green objects can be then sintered at a high temperature to densify ceramic walls. This approach can create three-dimensionally interconnected pores with tunable pore sizes, which are hardly obtainable using traditional manufacturing techniques (e.g., porogen leaching and sponge replication techniques) [13]. In addition, several approaches have been proposed to manufacture dual-scale porosity ceramics comprised of large pores surrounded by microporous ceramic walls. For example, micropores can be readily obtained by inducing necking between particles after partial sintering [27]. Two-stage freeze casting can create lamellar microstructures with interlamellar bridges [28]. The use of short fibers as a building block for freeze casting can create fibrous walls [29]. Freeze casting coupled with a carbothermal reduction process can create nanofibrous walls surrounding large lamellar pores [30,31]. In addition, ceramic/camphene (C_10_H_16_) suspensions can be frozen at room temperature because of the relatively high melting point of camphene (~48 °C) [32,33,34]. Thus, frozen ceramic compounds can be used as a feedstock for 3D printing, such as digital light processing [35] and UV-curing assisted 3D plotting [36,37]. These approaches can manufacture dual-scale porosity ceramics comprised of relatively large pores separated by porous ceramic walls. However, interconnectivity between pores created as the replica of camphene crystals and overall porosity need to be improved, in order to widen their applications.

Thus, we herein employed poly(methyl methacrylate) (PMMA) microspheres as a supplementary pore-forming agent in camphene-based freeze casting for the manufacture of dual-scale porosity ceramic structures. Basically, polymeric microspheres have been widely used to produce porous ceramics, since they can be readily removed by heat-treatment [38,39,40,41]. However, little attention has been paid to the utilization of polymeric microspheres in freeze casting presumably due to the potential segregation of polymeric microspheres with relative low density during freezing. We specially employed a PMMA polymer, since it is stable in molten camphene [42]. It should be noted that polystyrene (PS) is not applicable to camphene-based freeze casting because of its solubility in molten camphene [43]. In addition, PMMA microspheres with several microns were used, in order to induce effective rejection by growing camphene crystals without segregation. We examined how the addition of PMMA microspheres would affect the freezing behavior of alumina/camphene suspensions prepared at 60 °C and generation of dual-scale porosity. The effect of PMMA content on the overall porosity and compressive strength - of dual-scale porosity alumina ceramics was examined. In addition, the effect of camphene content at a given PMMA content was examined. In order to demonstrate the utility of alumina/camphene suspensions containing PMMA porogen, multi-scale porosity structures were also constructed using a 3D plotting technique.

## 2. Materials and Methods

### 2.1. Starting Materials

Table 1 summarizes the components of an alumina suspension prepared using camphene as a thermo-regulated phase separable vehicle (i.e., freezing vehicle) and poly(methyl methacrylate) (PMMA) microspheres as a porogen. All components were used as received. 

### 2.2. Freeze Casting of Alumina Suspensions

Alumina suspensions were prepared by mixing alumina particles, PMMA microspheres, and dispersant with molten camphene at 60 °C by ball-milling for 24 h. To control the overall porosity and porous structure of dual-scale porosity alumina objects, various PMMA contents (0 vol%, 20 vol%, 30 vol%, and 40 vol%) with respect to the alumina content were employed, while the same camphene content (70 vol%) with respect to the alumina/PMMA content was used for all suspensions (Table 2). In a similar way, two different camphene contents (60 vol% and 80 vol%) with the same PMMA content (30 vol%) were also prepared. 

**Figure 1:**
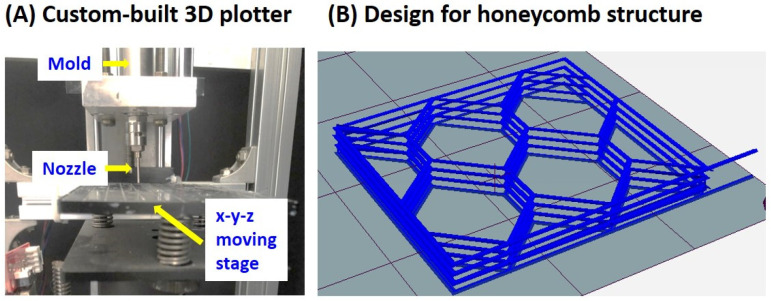
(**A**) Custom-built 3D plotter used to manufacture a honeycomb structure and (**B**) design of a honeycomb structure.

Prior to freeze casting, polyethylene (PE) molds with a diameter of ~6.3 mm were cooled at −20 °C, in order to induce the rapid solidification of alumina suspensions at room temperature. Alumina suspensions prepared at 60 °C were poured into the cool PE molds. After that, the solidified samples were removed from the PE molds. The diameter and height of the samples were ~6.3 mm and ~8.4 mm, respectively. In addition, honeycomb structures composed of periodic hexagonal macrochannels surrounded by dual-scale porosity alumina walls were constructed using a 3D plotting technique similar to the ceramic/camphene-based 3D extrusion process [44]. To this end, an alumina suspension was cast prepared at 60 °C into a metallic mold with an inner diameter of 10 mm, and then frozen at room temperature. The frozen feedstock was then extruded through a 1 mm diameter at a constant extrusion speed of 120 mm/min (Figure 1A). The extruded filaments were then deposited at a constant speed of 110 mm/min according to a predetermined build path (Figure 1B). 

The green samples were freeze-dried to remove camphene crystals. Thereafter, the porous green bodies were slowly heat-treated, particularly in the temperature range of 400 °C–600 °C, in order to carefully remove PMMA microspheres, and then finally sintered at 1550 °C for 3 h, in order to densify alumina walls. 

### 2.3. Porous Structure and Microstructure Evaluations 

The sintering shrinkages of dual-scale porosity alumina structures produced using various PMMA and camphene contents were computed by measuring their diameters before and after sintering. The densities of the sintered objects were calculated by measuring their mass and volume. Relative density and overall porosity were computed by considering the theoretical density of alumina (3.97 g/cm^3^). Porous structures and microstructures of the green and sintered objects were characterized by field emission scanning electron microscopy (FE-SEM; JSM-6701F, JEOL Techniques, Tokyo, Japan). 

### 2.4. Compressive Strength Tests

Mechanical properties of the dual-scale porosity alumina structures produced using various PMMA and camphene contents were characterized using a universal testing machine (Instron 5582, Instron Corp., Canton, MA, USA). Samples of ~5.2 mm in diameter and ~7.0 mm height were compressed at a constant crosshead speed of 1 mm/min. During the tests, compressive loads were recorded as a function of displacement. Compressive strengths of the samples were computed by considering their peak load and cross-sectional area. Five samples were tested for each condition, in order to obtain the mean value and deviation. 

## 3. Results and Discussion

### 3.1. Effect of PMMA Addition on Pore Structure and Microstructure of Green Objects

In this study, we investigated the utility of thermo-regulated phase separable alumina/camphene suspensions containing PMMA microspheres as a supplementary pore-forming agent for the production of multi-scale porosity structures. PMMA polymer was specially employed, since it would not dissolve in molten camphene [42] unlike polystyrene (PS) [43]. Fundamentally, our approach adopts the principle of camphene-based ceramic freeze casting [33]. More specifically, when placed below the melting point of camphene (~42 °C), alumina suspensions can undergo phase separation, resulting in a three-dimensional network of camphene crystals, surrounded by walls composed of alumina particles and PMMA microspheres. Thus, large interconnected dendritic pores can be created by the removal of camphene crystals via freeze-drying, while relatively small spherical pores can be generated in alumina walls by the removal of PMMA microspheres via heat treatment [38,39,40]. 

**Figure 2:**
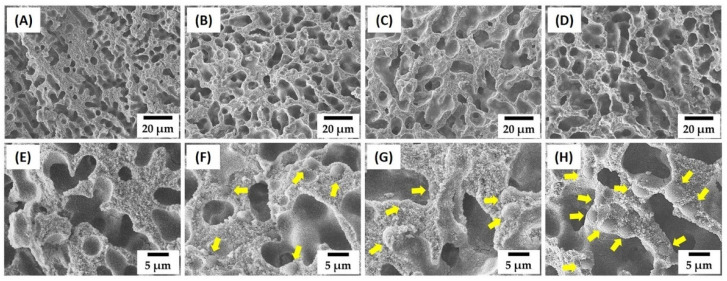
Representative FE-SEM images of green objects produced using different PMMA contents: 0 vol% (**A**,**E**), 20 vol% (**B**,**F**), 30 vol% (**C**,**G**), and 40 vol% (**D**,**H**). The yellow arrows indicate PMMA microspheres in alumina walls.

To examine the effect of PMMA addition on the development of dual-scale porosity structures, four kinds of alumina/camphene suspensions with various PMMA contents (Table 2)—0 vol%, 20 vol%, 30 vol%, and 40 vol% with respect to the alumina content—were prepared at 60 °C. The prepared suspensions were then cast into cool PE molds, followed by solidification at room temperature. After freeze-drying for the removal of camphene crystals, the porous structures and microstructure of the green samples were characterized by FE-SEM, as shown in Figure 2A–H. Without PMMA addition, the sample showed the typical porous structure of a freeze-cast ceramic body (Figure 2A). Interconnected pores with high aspect ratios were created as the replica of camphene crystals that had grown dendritically along the direction of heat conduction [43,45]. 

Alumina particles were highly concentrated (Figure 2E), which would result in highly densified alumina walls after sintering. In addition, all samples produced using PMMA addition showed highly porous structures (Figure 2B–D). This finding suggests that PMMA addition did not hinder the dendritic growth of camphene crystals during freezing. However, interestingly, PMMA addition resulted in larger dendritic pores, and the size increased slightly with the increase in PMMA content. Although a further study is required, it is reasonable to suppose that the size of camphene crystals is limited when the force generated by packed particles starts to exceed the force generated by the growing crystal to push particles [46]. Thus, PMMA microspheres with much lower density and larger size (a size range of 0.3–6.55 μm used in this study) than alumina particles could be more easily pushed by growing camphene crystals. This resulted in larger dendritic pores. All samples produced with PMMA addition showed highly concentrated walls, composed of alumina particles surrounding PMMA microspheres that are indicated by the yellow arrows (Figure 2F–H). More PMMA microspheres were observed for the higher initial PMMA content added into suspensions. This finding suggests that, during the dendritic growth of camphene crystals, PMMA microspheres added into alumina suspensions could be effectively pushed with alumina particles without segregation. 

### 3.2. Dual-Scale Pore Structures of Sintered Objects

Green objects produced using various PMMA contents (0 vol%, 20 vol%, 30 vol%, and 40 vol%) were carefully heat-treated particularly at the temperature range of 400 °C–600 °C to remove PMMA microspheres, followed by sintering at 1550 °C for 3 h. Sintering shrinkage decreased from 17.8 ± 0.4% to 15.3 ± 0.7 vol% with the increase in PMMA content from 0 vol% to 30 vol% (Table 3). However, a very small change was observed for higher PMMA content (40 vol%). This was attributed to the reduction in the fraction of alumina walls required for densification. 

**Figure 3:**
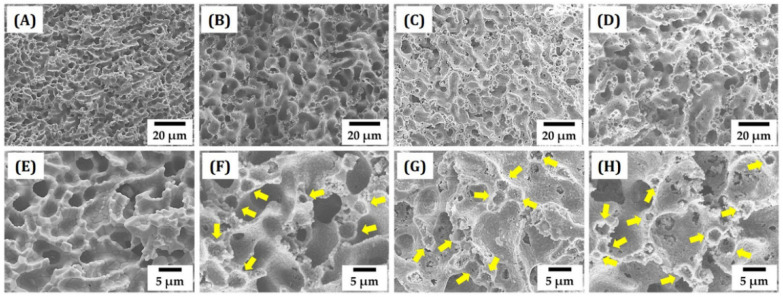
Representative FE-SEM images of dual-scale porosity structures produced using different PMMA contents: 0 vol% (**A**,**E**), 20 vol% (**B**,**F**), 30 vol% (**C**,**G**), and 40 vol% (**D**,**H**). The yellow arrows indicate the pores created by the removal of PMMA microspheres via heat treatment.

Figure 3A–H show representative FE-SEM images of sintered objects. Without PMMA addition, the sample showed the typical porous structure of a freeze-cast ceramic [33]. That is, a 3-dimensional network of dendritic pores was created as a replica of camphene crystals (Figure 3A). In addition, the alumina walls were almost fully densified (Figure 3E), which is attributed to the high packing density of alumina particles after phase separation. On the other hand, with PMMA addition, the objects showed two types of pores—relatively large dendritic pores and small pores in alumina walls (Figure 3B–D). The creation of spherical pores by the removal of PMMA microspheres is more clearly visible in Figure 3F–H. No noticeable defects were observed in the alumina walls. A higher PMMA content resulted in a more porous structure in the alumina walls. Such small pores are expected to enhance pore interconnectivity, which is useful for some applications. For examples, when used as bone scaffolds, they can provide excellent bone regeneration ability with reasonably high mechanical properties [19,20]. This finding suggests that a dual-scale porosity alumina structure could be constructed using a combination of camphene and PMMA microspheres. In other words, camphene as a thermo-regulated phase separable vehicle can construct a 3D dendritic pore network, while PMMA microspheres as a pore-forming agent can create small pores in the alumina walls. 

### 3.3. Total Porosities of Dual-Scale Porosity Structures

**Figure 4:**
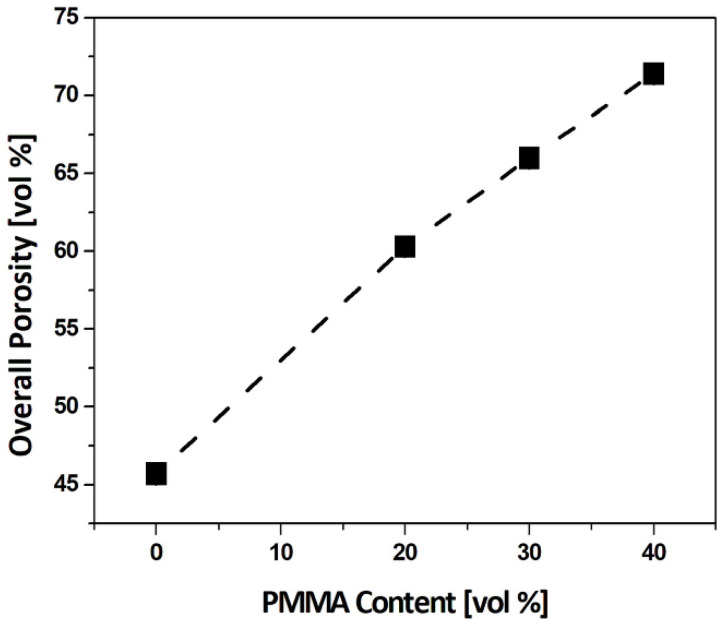
Total porosities of dual-scale porosity structures as a function of PMMA content. The dashed line represents the computed relationship between the total porosity and initial PMMA content.

Figure 4 show the total porosities (*P*_T_) of the dual-scale porosity structures produced using different PMMA contents (0 vol%, 20 vol%, 30 vol%, and 40 vol%), computed by measuring their weight and volume. Total porosity increased from 45.7 ± 0.5 vol% to 71.4 ± 0.5 vol% with an increase in PMMA content from 0 vol% to 40 vol%. A linear relationship between the total porosity and initial PMMA content, marked by the dashed line, can be observed. This finding suggests that most of the PMMA microspheres could be effectively pushed by camphene crystals without engulfment within camphene crystals, and thus *P*_P_ can be readily tailored by adjusting the initial PMMA content in an alumina suspension, resulting in tunable *P*_T_. 

### 3.4. Compressive Strengths of Dual-Scale Porosity Structures

**Figure 5:**
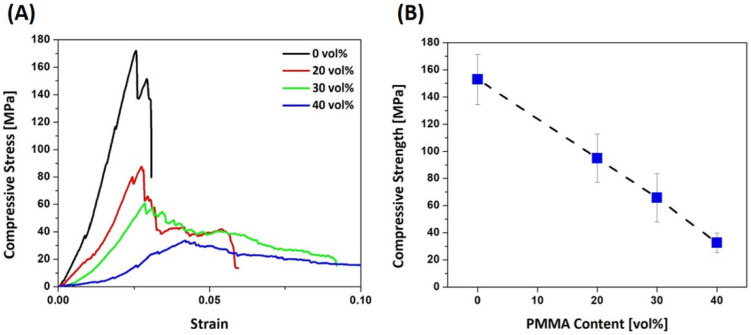
(**A**) Representative compressive stress versus strain responses of the dual-scale porosity alumina structures produced using different PMMA contents (0 vol%, 20 vol%, 30 vol%, and 40 vol%) and (**B**) measured compressive strengths as a function of PMMA content.

The mechanical properties of dual-scale porosity alumina structures produced using different PMMA contents (0 vol%, 20 vol%, 30 vol%, and 40 vol%) were measured by compressive strength tests. Figure 5A shows the representative compressive stress versus the strain responses of samples. Without PMMA addition, the sample showed a rapid increase in stress with an increase in strain. After reaching the maximum value, it rapidly decreased, indicating the brittle fracture of the alumina walls [47,48]. The sample produced using the lowest PMMA content (20 vol%) showed a similar stress–strain curve. However, even after the maximum value, a retention of noticeable stress was observed. This tendency became more obvious with an increase in PMMA contents to 30 vol% and 40 vol%. This was attributed to the local fractures of the porous alumina walls. More specifically, some of the porous alumina walls were first fractured instead of an entire fracture occurring. Thus, the remaining alumina walls could to a certain extent withstand additional loads. Compressive strengths, computed from the peak loads observed, decreased from 152.9 ± 18.3 MPa to 32.6 ± 7.2 MPa with an increase in PMMA content from 0 vol% to 40 vol%, as shown in Figure 5B. These values are comparable to, or even higher than, those obtained for the porous alumina produced using camphene-based freeze casting [45,49]. This finding suggests the utility of dual-scale porosity structures compared to uniform porous structures. 

### 3.5. Control over Dual-Scale Pore Structures by Camphene Content

**Figure 6:**
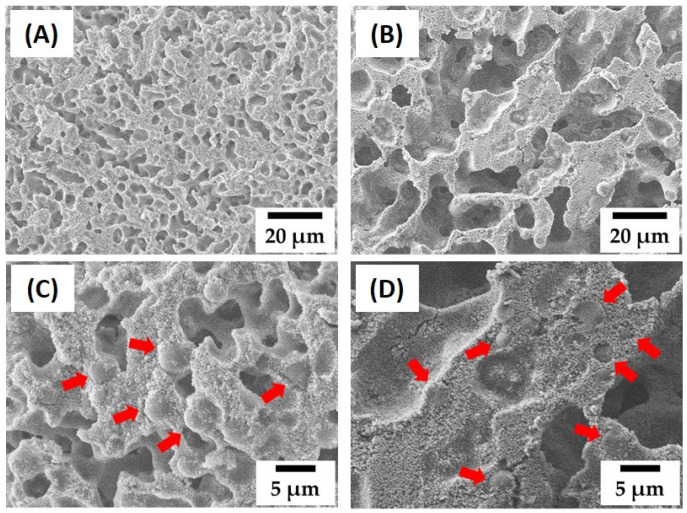
Representative FE-SEM images of green alumina samples produced using different camphene contents: 60 vol% (**A**,**C**) and 80 vol% (**B**,**D**). The red arrows indicate the PMMA microspheres.

To further tailor the porous structure and compressive strength of dual-scale porosity alumina structures, we examined the effect of camphene content, while the same PMMA content (30 vol% with respect to the alumina) was used. Figure 6A–D show representative FE-SEM images of green samples produced using different camphene contents (60 vol% and 80 vol%). Both samples showed highly porous structures without any notable defects in alumina walls (Figure 6A,B), while MMA microspheres, indicated by the red arrows, were well dispersed in the alumina walls (Figure 6C,D). However, a higher camphene content resulted in a higher pore fraction and larger pore size, which is often the case with camphene-based freeze casting [33]. 

**Figure 7:**
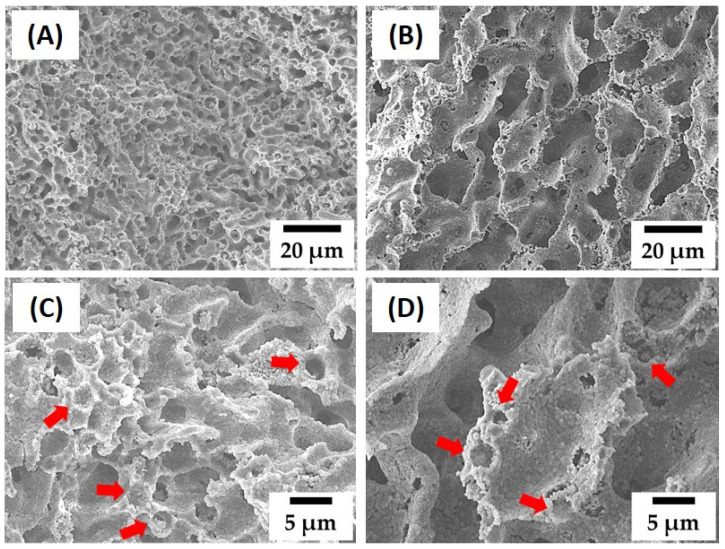
Representative FE-SEM images of the sintered alumina samples with a dual-scale porosity structure produced using different camphene contents: 60 vol% (**A**,**C**) and 80 vol% (**B**,**D**). The red arrows indicate the pores created by the removal of the PMMA microspheres via heat treatment.

Figure 7A–D show representative FE-SEM images of sintered alumina samples with a dual-scale porosity structure produced using different camphene contents (60 vol% and 80 vol%). Both samples showed highly porous structures (Figure 7A,B) composed of two kinds of pores, i.e., three-dimensional dendritic pores and small spherical pores, indicated by red arrows (Figure 7C,D).

**Figure 8:**
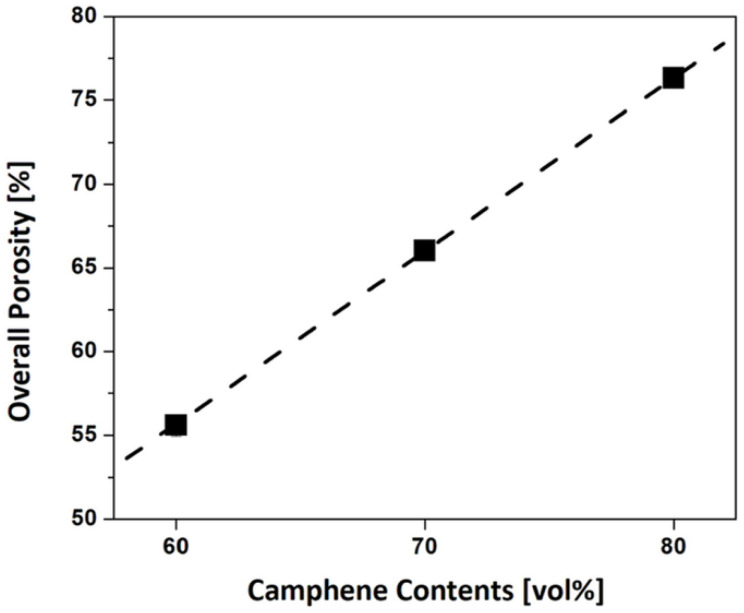
Total porosities of the dual-scale porosity alumina structures as a function of camphene content.

The total porosities of samples produced using camphene contents of 60 vol% and 80 vol% were 55.6 ± 0.7 vol% and 76.3 ± 0.3 vol%, respectively. To interpret the effect of camphene content on the total porosity of the dual-scale porosity structures, Figure 8 plots the total porosities obtained using different camphene contents (60 vol%, 70 vol%, and 80 vol%). The total porosity (*P*_T_) was observed to increase almost linearly with an increase in camphene content (V_c_), which can be described as follows:

*P*_T_ = (1.03 × V_c_) − 6.34 (1)

This linear relationship was attributed to the fact that since they used the same PMMA content (30 vol%), they could have a similar fraction of spherical pores, while the fraction of dendritic pores could increase linearly with an increase in camphene content. This finding suggests that total porosity can be readily tuned by adjusting the camphene content at a constant PMMA content.

**Figure 9:**
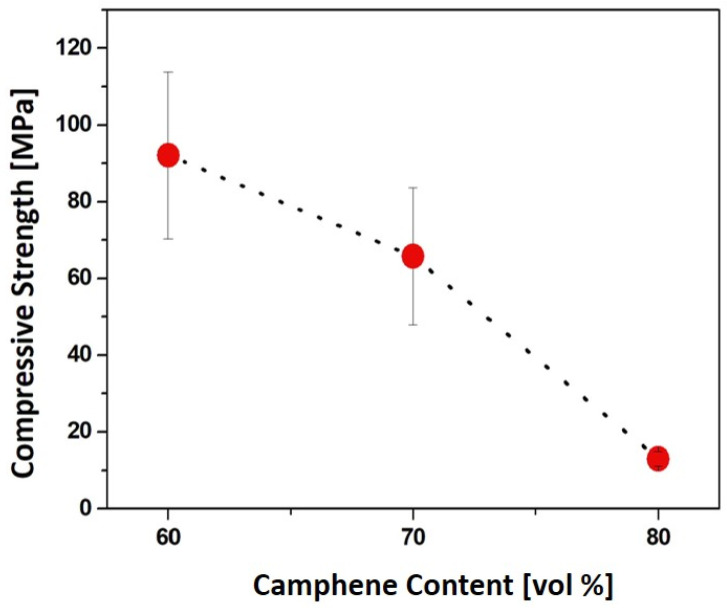
Measured compressive strengths as a function of camphene content.

Compressive strengths, computed from the peak loads observed, decreased from 92.1 ± 21.7 MPa to 13.0 ± 1.9 MPa with an increase in camphene content from 60 vol% to 80 vol%, as shown in Figure 9. 

### 3.6. Overall Relationship between Total Porosity and Compressive Strength 

**Figure 10:**
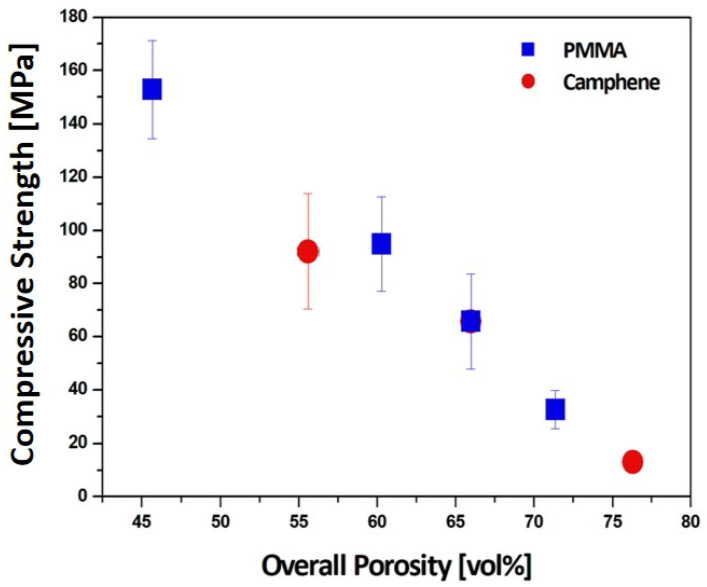
Compressive strengths of dual-scale porosity alumina structures produced using different PMMA and camphene contents as a function of total porosity. The blue and red symbols represent the compressive strengths obtained from different PMMA and camphene contents, respectively.

The compressive strengths of the dual-scale porosity structures produced using camphene contents of 60 vol% and 80 vol% were 92.1 ± 21.7 MPa and 13.0 ± 1.9 MPa, respectively. To demonstrate the ability to tailor the total porosity and compressive strengths of the dual-scale porosity alumina structures produced using our approach, Figure 10 plots the compressive strengths obtained using different PMMA (20 vol%, 30 vol%, and 40 vol%) and camphene contents (60 vol%, 70 vol%, and 80 vol%) as a function of total porosity. As expected, the compressive strength decreased with an increase in total porosity. However, this reduction is not exponential [50], presumably due to the creation of dual-scale porosity. In addition, the sample with the lowest porosity (~55.6 vol%) obtained using a camphene content of 60 vol% with a PMMA content of 30 vol% showed a slightly lower compressive strength than that obtained using a higher camphene content of 70 vol% with a lower PMMA content of 20 vol%. This was attributed to the dual-scale porosity structures being more likely to fracture by large dendritic pores at given porosities. Thus, it is reasonable to suppose that the creation of dual-scale porosity can be one of the most promising approaches for the achievement of high porosities with reasonably high mechanical properties. Note that the measured compressive strengths are in the range of ~94.9–32.6 MPa, which is comparable to that of natural bones [2], and thus dual-scale porosity alumina structures can be used as bone scaffolds.

### 3.7. Feasibility as Feedstock for 3D Printing of Multi-Scale Porosity Structures 

**Figure 11:**
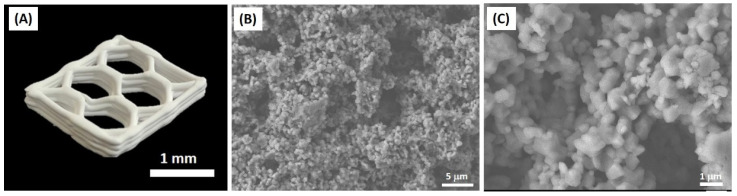
(**A**) Representative optical image of a multi-scale porosity object with a honeycomb structure and FE-SEM images showing elongated pores created by the removal of camphene crystals at different magnifications (**B**,**C**).

We examined the feasibility of an alumina/camphene suspension containing PMMA porogen as a feedstock for a 3D plotting process, in order to construct multi-scale porosity structures. To this end, frozen feedstock was extruded through a 1 mm diameter nozzle, and the extruded filaments were then deposited using our custom-made 3D plotter according to predetermined build paths. As a model, a honeycomb structure was manufactured. It was observed that the frozen feedstock, composed of camphene dendrites surrounded by alumina particles with PMMA microspheres, could be extruded without difficulty, owing to the good extrudability of camphene with soft wax-like behavior [44,51]. The produced sample showed a well-defined honeycomb structure even after sintering at 1550 °C for 3 h (Figure 11A). In addition, filaments were well bonded together without any noticeable defects. Two types of pores—relatively large and small pores due to the removal of camphene crystals and PMMA microspheres—were observed (Figure 11B,C). This new approach can allow for the creation of three different types of pores—periodic hexagonal macrochannels by 3D plotting process, highly elongated pores by camphene crystals, and small spherical pores by PMMA microspheres. This finding suggests that a variety of multi-scale porosity structures can be constructed.

## 4. Conclusions

Dual-scale porosity alumina structures could be constructed using a combination of camphene as a thermo-regulated phase separable vehicle and PMMA microspheres as a pore-forming agent in alumina suspensions, which could create three-dimensionally interconnected large dendritic pores and relatively small spherical pores in alumina walls, respectively. In addition, the overall porosity and compressive strength could be readily tailored simply by adjusting the PMMA and camphene contents. Reasonably high compressive strengths in the range of ~94.9–32.6 MPa could be obtained for high overall porosities (~60.3 vol%–~71.4 vol%) owing to the creation of dual-scale porosity. In addition, these newly formulated alumina suspensions could be utilized as a feedstock to manufacture multi-scale porosity structures using a 3D plotting process.

## Figures and Tables

**Table 1:** Components of an alumina suspension prepared using camphene as a thermo-regulated phase separable vehicle (i.e., freezing vehicle) and poly(methyl methacrylate) (PMMA) microspheres as a porogen.

Component	Material (Supplier)
**Ceramic Powder**	Alumina (Kojundo Chemical Co., Ltd., Sakado, Japan)
**Freezing Vehicle**	Camphene (Sigma-Aldrich, St. Louis, MO, USA)
**Porogen**	Poly(methyl methacrylate) (PMMA) (Sunjin Beauty Science, Ansan-si, Korea)
**Dispersant**	Hypermer KD-4 (UniQema, Everburg, Belgium)

**Table 2:** Compositions of various alumina suspensions used to produce multi-scale porosity alumina structures with controlled porous structures.

	Camphene [vol%]	PMMA [vol%]	Alumina [vol%]	KD4 [vol%]
**PMMA Contents**	70.00	0.00	27.57	2.43
	70.00	6.00	21.91	2.09
	70.00	9.00	19.08	1.92
	70.00	12.00	16.25	1.75
**Camphene Contents**	60.00	12.00	25.44	2.56
	80.00	6.00	12.72	1.28

**Table 3:** Sintering shrinkages of dual-scale porosity structures produced using different PMMA contents (0 vol%, 20 vol%, 30 vol%, and 40 vol%).

PMMA Content [vol%]	0	20	30	40
**Sintering shrinkage [%]**	17.8 ± 0.4	16.2 ± 0.7	15.3 ± 0.7	15.6 ± 1.0

## Data Availability

Not applicable.
